# Physiological Mechanisms of the Enhanced UV-B Radiation Triggering Plant-Specific Peroxidase-Mediated Antioxidant Defences

**DOI:** 10.3390/antiox14080957

**Published:** 2025-08-04

**Authors:** Yijia Gao, Ling Wei, Chenyu Jiang, Shaopu Shi, Jiabing Jiao, Hassam Tahir, Minjie Qian, Kaibing Zhou

**Affiliations:** 1 Sanya Institute of Breeding and Multiplication, Hainan University, Sanya 572025, China; 2 Key Laboratory of Quality Regulation of Tropical Horticultural Crop in Hainan Province, School of Tropical Agriculture and Forestry, Hainan University, Haikou 570228, China

**Keywords:** plant-specific peroxidase, enhanced UV-B radiation, antioxidant defences, lignin, hormone, POD multigene family

## Abstract

In this study, an artificially simulated enhanced UV-B radiation treatment of 96 kJ/m^2^·d^−1^ was applied with natural sunlight as the control. By observing changes in biological tissue damage, peroxidase (POD) enzyme activity, and hormone content, combined with transcriptome analysis and quantitative fluorescence PCR validation, this study preliminarily elucidated the physiological mechanisms of plant-specific peroxidase (POD) in responding to enhanced UV-B radiation stress. Enhanced UV-B treatment significantly inhibited biological tissue growth, particularly during the rapid growth stage. At this stage, the treatment exhibited higher malondialdehyde (MDA) content, indicating increased oxidative stress due to the accumulation of reactive oxygen species (ROS). Despite the inhibition in growth, the treatment showed improvements in the accumulation of organic nutrients as well as the contents of abscisic acid (ABA), salicylic acid (SA), and methyl jasmonate (MeJA). Additionally, an increase in POD activity and lignin content was observed in the treatment, especially during the middle period of the rapid growth period. Transcriptome analysis revealed that two POD multigene family members, *LOC123198833* and *LOC123225298*, were significantly upregulated under enhanced UV-B radiation, which was further validated through qPCR. In general, enhanced UV-B radiation triggered a defence response in biological tissue by upregulating POD genes, which can effectively help to scavenge excess ROS.

## 1. Introduction

Light is a vital environmental factor that regulates plant growth and development. Beyond serving as the primary energy source for photosynthesis, light influences a wide range of physiological and developmental processes throughout the plant life cycle, including seed germination, seedling photomorphogenesis, stem elongation, lateral root formation, shade avoidance, and circadian rhythm regulation [1,2]. However, light can also act as an environmental stressor, particularly in the form of high-intensity light and ultraviolet (UV) radiation within the 200–400 nm wavelength range, which can negatively affect plant physiology and development [3]. Over the past few decades, human activities—such as the increased use of chlorofluorocarbons (CFCs) and vehicle emissions—have released significant amounts of bromine- and chlorine-containing compounds into the atmosphere. These substances contribute to ozone layer depletion, resulting in increased levels of ultraviolet-B (UV-B) radiation (280–320 nm) reaching the Earth’s surface. This phenomenon is referred to as enhanced UV-B radiation [4,5]. It has been reported that moderate doses of enhanced UV-B radiation play an important ecological role in regulating the growth and development of many plants, while high doses may cause damage to plants at the cellular and molecular levels [6,7].

Enhanced ultraviolet-B (UV-B) radiation triggers a range of physiological responses in plants, primarily through the overproduction of reactive oxygen species (ROS), leading to oxidative stress. These reactive species, including superoxide anion (O_2_•^−^), hydroxyl radical (•OH), hydrogen peroxide (H_2_O_2_), and singlet oxygen (^1^O_2_), can damage cellular components such as membrane lipids, nucleic acids, and proteins [8]. To mitigate the detrimental effects of reactive oxygen species (ROS) generated under enhanced UV-B radiation stress, plants have developed complex antioxidant defence systems comprising both enzymatic and non-enzymatic antioxidant mechanisms [9]. Among the enzymatic antioxidants, peroxidases (PODs) play a crucial role. These enzymes catalyse the oxidation of various substrates using hydrogen peroxide (H_2_O_2_) or other organic peroxides (ROOH) as electron acceptors [10]. Through these oxidation reactions, PODs contribute to critical physiological processes, including signal transduction and lignin biosynthesis, thereby enhancing plant tolerance to both biotic and abiotic stresses [11].

PODs can broadly be classified into heme and non-heme enzymes, depending on the presence of a heme prosthetic group [12]. Based on phylogenetic and functional classifications, heme peroxidases are grouped into three major superfamilies: non-animal peroxidases, the peroxidase–cyclooxygenase superfamily, and a miscellaneous group. The non-animal peroxidases are further divided into four subclasses: Class I (prokaryotic peroxidases), Class II (fungal secretory peroxidases), Class III (plant-specific peroxidases), and other specialised non-animal peroxidases [13]. Notably, Class III peroxidases are unique to plants and show significant gene family expansion. Numerous Class III POD genes have been identified across major crop species, including 669 members in wheat (*Triticum aestivum* L.), 155 in maize (*Zea mays* L.), and 117 in rice (*Oryza sativa* L.) [14]. This expansion highlights their vital role in plant adaptation and stress resilience [14].

PODs are widely involved in various biological processes, including hormone synthesis and degradation, fruit development, cell wall metabolism, and defence against pathogenic microorganisms [11]. However, plant-specific peroxidases are associated with normal plant morphogenesis and play vital roles in plant growth and development, such as lignin biosynthesis, seed germination, cell elongation, auxin metabolism, deposition of cell wall cross-linking compounds, and plant hormone metabolism. In addition to their developmental roles, the expression of plant-specific peroxidases structural genes is also strongly induced under stress conditions. As key protective enzymes, they contribute significantly to plant defence against a range of adverse environmental factors, such as pathogen attack, drought, cold, heavy metal toxicity, and mechanical injury [14,15].

PODs are also critically involved in the plant response to enhanced ultraviolet-B (UV-B) radiation. Numerous studies have demonstrated that elevated UV-B levels can significantly increase POD activity, thereby enhancing antioxidant capacity and alleviating oxidative stress. For instance, buckwheat (*Fagopyrum esculentum* M.) and tea plants (*Camellia sinensis*) exhibit higher POD activity under UV-B, which lowers harmful reactive oxygen species like H_2_O_2_ and O_2_^−^ [16,17]. In rice seedlings, exogenous melatonin under enhanced UV-B conditions further stimulated POD activity, promoted ROS scavenging, and provided cellular protection [18]. Lettuce and cucumber also show increased POD activity and gene expression under UV-B, helping to degrade accumulated H_2_O_2_ and mitigate stress damage [19,20,21]. Similarly, enhanced UV-B radiation has been shown to elevate POD activity in other plants such as Beta vulgaris, cucumber, and Hibiscus rosa-sinensis [22]. Collectively, these studies underscore the vital role of PODs in regulating redox balance and improving plant tolerance to UV-B-induced oxidative stress.

**Figure 1:**
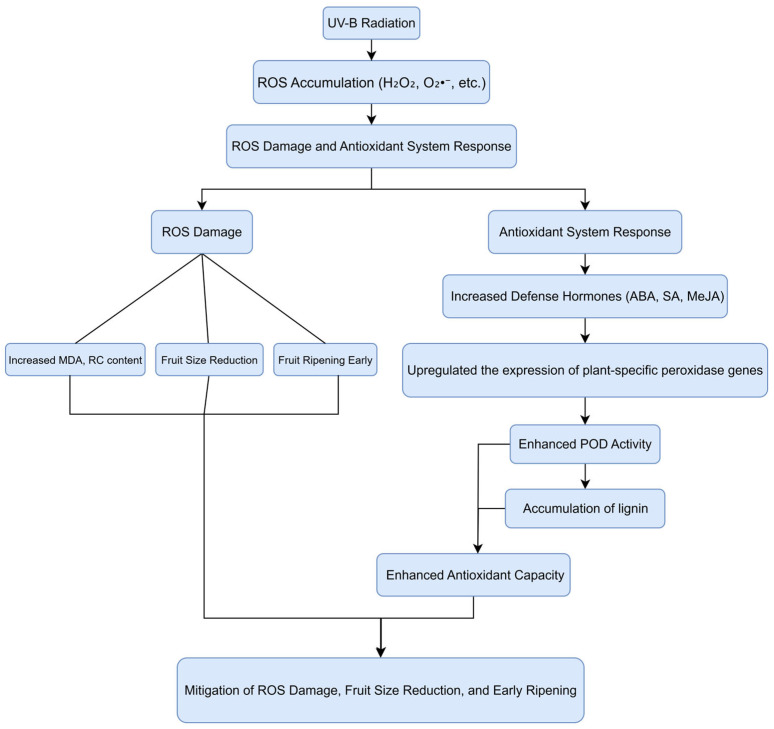
Physiological and biochemical processes in mango under UV-B radiation.

Mango (*Mangifera indica* L.), a member of the *Mangifera* genus and the Anacardiaceae family, is widely cultivated in over 90 countries and holds a high economic importance in tropical and subtropical regions [23]. In comparison to high-latitude regions, low-latitude areas are projected to experience a more pronounced increase in surface-level UV-B radiation. As a result, enhanced UV-B exposure may emerge as a significant abiotic stressor for mango cultivation. Previous research conducted by our team demonstrated that simulated enhanced UV-B radiation led to a decline in mango fruit external quality, increased membrane lipid peroxidation in the fruit pulp cells, and elevated POD activity during the initial stages of exposure [24]. This study aims to further investigate the physiological mechanisms of the response of plant-specific peroxidases in ‘Tainongyihao’ mango pulp in response to enhanced UV-B radiation stress. The research overview was shown in Figure 1, and the findings would lay a theoretical foundation for breeding enhanced UV-B radiation-resistant mango varieties and developing cultivation techniques to mitigate enhanced UV-B radiation stress.

## 2. Materials and Methods

### 2.1. Experiment Site and Plant Material

The experiment was conducted in a mango orchard located in Shengchang Village, Haitang District, Sanya City, Hainan Province (18°25′ N, 109°46′ E). The orchard in Shengchang Village serves as a commercial plantation. The site experiences an average annual precipitation of approximately 1700 mm and a mean annual temperature of around 25 °C. The soil type is classified as brick-red sandy soil, and the trees were planted at a spacing of 4 m × 5 m. Ten vigorous and uniformly grown 17-year-old ‘Tainongyihao’ mango trees were selected as experimental material.

### 2.2. Methods

#### 2.2.1. Experimental Design

Previous research by our research group has shown that artificial simulation of enhanced UV-B radiation at 96 kJ.m^−2^·d^−1^, equivalent to a 15% increase on average in natural UV-B radiation, significantly reduced the yield and quality of ‘Tainongyihao’ mango trees [24]. Therefore, the treatment group in the present study was subjected to simulated enhanced UV-B radiation using four 40 W UV-B lamps (λ = 313 nm, Beijing Electric Light Source Research Institute, Beijing, China) arranged crosswise and evenly spaced at a height of 40 cm above the tree canopy. The control group was exposed only to natural sunlight. The experiment was designed using single-tree plots with five biological replicates per treatment [25]. Field trials were carried out for two consecutive fruit growth cycles: from 16 November 2023 (30 days after anthesis) to 6 January 2024 (91 days after anthesis), and from 10 March 2024 (30 days after anthesis) to 11 May 2024 (92 days after anthesis). During these periods, the UV-B lamps were operated daily from sunrise to sunset, with treatments paused on cloudy or rainy days.

#### 2.2.2. Sampling and Sample Pre-Treatment

Five representative, medium-sized fruits were selected from the middle portion of the canopy periphery of each experimental tree to serve as reference samples. Fruit collection began at the initiation of the enhanced UV-B radiation treatment and continued at 10-day intervals thereafter. Sampling was conducted based on the size and colouration stage of the reference fruits. Fruit samples were processed in the field and then stored at −80 °C in an ultra-low temperature freezer for future analysis.

#### 2.2.3. Detection of Pulp Quality Indicators

The soluble sugar content (TSS) in pulp was determined using the anthrone colourimetric method. Total organic acid content (TA) was measured with a Brix-acid meter (PAL-BX/ACID15; ATAGO, Tokyo, Japan), and the sugar-to-acid ratio (TSS/TA) was calculated. Ascorbic acid (ASA) content was determined using a reagent kit (Catalogue No: ASA-2-W, Suzhou Comin Biotechnology Co. Ltd., Suzhou, China), following the protocols recommended by the manufacturer.

#### 2.2.4. Detection of Malondialdehyde Content and Relative Conductivity of Pulp

Malondialdehyde (MDA) content in the fruit pulp was quantified using assay kits (Catalogue No: G0109F, Suzhou Grace Biotechnology Co. Ltd., Suzhou, China), following the manufacturer’s instructions. However, relative conductivity (RC) was measured according to the method described by Deshmukh [26].

#### 2.2.5. Detection of Hormone Content in the Pulp

The contents of abscisic acid (ABA), methyl jasmonate (MeJA), and salicylic acid (SA) were measured using ELISA test kits (Catalogue No: YJ077235, YJ132940, YJ077224, Shanghai Enzyme-Linked Biotechnology Co., Ltd., Shanghai, China).

#### 2.2.6. Detection of POD Activity and Lignin Content

For the corresponding samples, the POD activity and lignin content of the fruit pulp were tested. POD activity was measured using an ELISA test kit (Catalogue No: KT5058-A, Keter Biotechnology Co., Ltd., Yancheng, China), and lignin content was determined using a lignin content kit (Catalogue No: MZS-2-G, Suzhou Comin Biotechnology Co., Ltd., Suzhou, China). In addition, units of POD activity were defined as one unit of enzyme viability per tissue per minute of A470 change of 0.01 per mL of reaction system.

#### 2.2.7. Screening and qPCR Verification of Differentially Expressed Genes

Based on the transcriptome data [27] and bioinformatics analysis of the POD multigene family [28] previously published by our team (the accession number for the mango transcriptome data is PRJNA1245353 in NCBI database), six high-expression POD gene family members belonging to plant-specific peroxidases were selected as key research targets in 2023 and 2024 (Table 1). Specific primers for qPCR were designed using the NCBI Primer-BLAST online tool (https://www.ncbi.nlm.nih.gov/tools/primer-blast/ accessed on 1 December 2024), and the primer synthesis was carried out by Bio Sune Biotechnology (Shanghai) Co., Ltd. (Shanghai, China). Total RNA was extracted from mango fruit pulp using the Steady Pure Plant RNA Extraction Kit (Catalogue No: AG21024, Accurate Biology Co., Ltd., Changsha, China). Reverse transcription was conducted with the Evo M-MLV Reverse Transcription PreMix Kit Ver. 2 (Catalogue No: AG11728, Accurate Biology Co. Ltd., Changsha, China) on a BIO-RAD T100FM Thermal Cycler, following the manufacturer’s protocols. Quantitative PCR amplification was carried out using the SYBR^®^ Green Premix Pro Taq HS qPCR Kit (Catalogue No: AG11701, Accurate Biology Co., Ltd., Changsha, China) on a qTOWER3 Real-Time PCR System (Analytik Jena AG, Jena, Germany). The mango Actin gene served as the internal reference, and relative gene expression levels were calculated by the 2^−ΔΔCt^ method. Primer sequences are listed in Table 1.

#### 2.2.8. Statistical Analysis

Graphs were generated using GraphPad Prism 9.5, and statistical analyses were conducted with SPSS 27.0 software. ANOVA was used to assess significant differences among the different periods of the treatment and control groups, respectively (*p* < 0.05 was considered significant; *p* < 0.01, highly significant, and the same as below). Multiple comparisons among different time points within treatment and control groups, respectively, were performed using Duncan’s multiple range test. Independent *t*-tests were applied to evaluate differences between the treatment and control groups at each corresponding time point. Multivariate linear correlation analysis among fruit pulp hormone levels, POD gene family expression, POD enzymatic activity, and lignin content was conducted using an online statistical analysis platform (https://www.chiplot.online/ accessed on 18 May 2025).

## 3. Results

### 3.1. Effect of Enhanced UV-B Radiation Treatment on Mango Fruit Size and Peel Colouration

**Figure 2:**
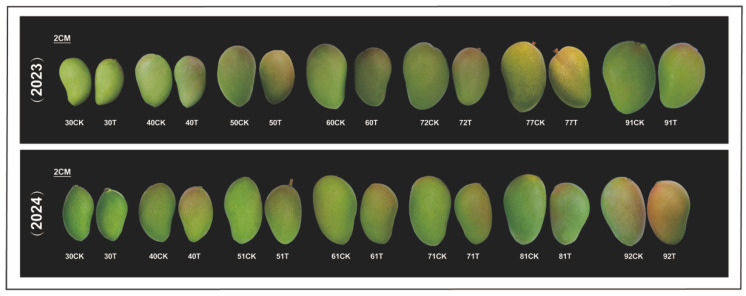
Effect of enhanced UV-B radiation treatment on mango fruit size and peel colouration. CK represents the control group; T represents the enhanced UV-B treatment.

The effects of the treatment on fruit size and peel colouration are shown in Figure 2. From 40 days after anthesis onward, treated fruits were significantly smaller than those of the control. In the later stages of fruit development, the peel of treated fruits turned yellow and developed colouration earlier than the control. These results indicated that the treatment suppressed fruit enlargement and accelerated fruit maturation.

### 3.2. Effect of Enhanced UV-B Radiation Treatment on the Main Nutritional and Flavour Qualities of Mango Pulp

**Figure 3:**
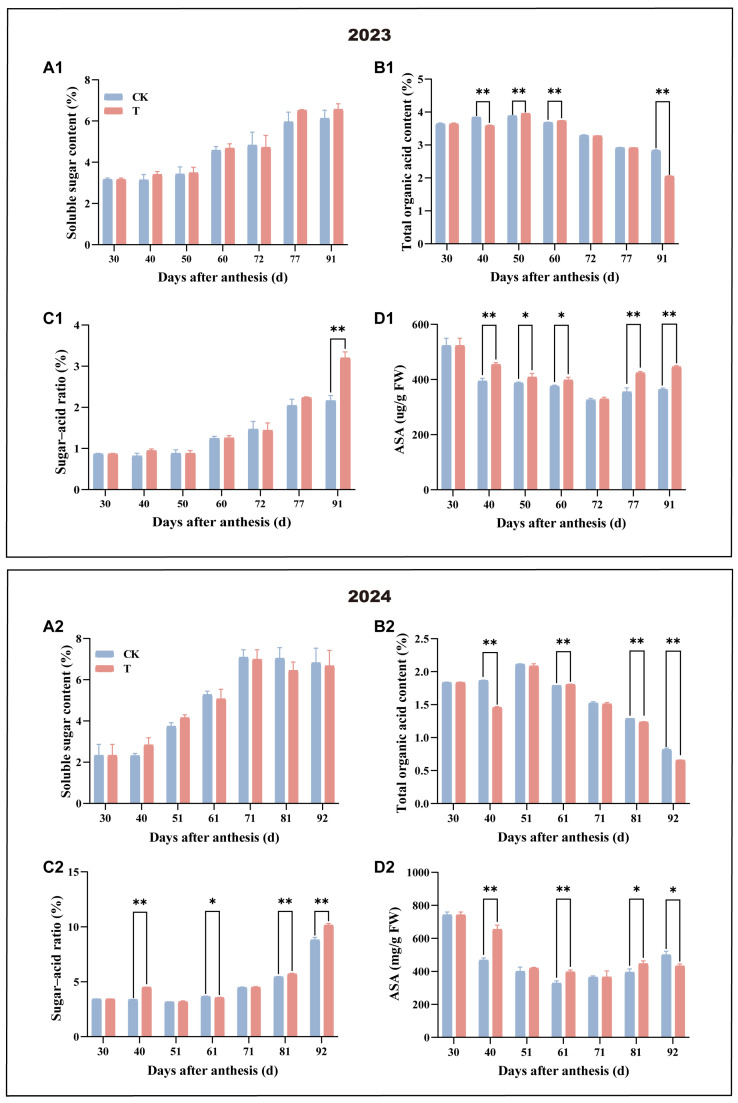
Effect of enhanced UV-B radiation treatment on mango fruit pulp. (**A1**,**A2**) Soluble sugar content; (**B1**,**B2**) total organic acid content; (**C1**,**C2**) sugar–acid ratio; and (**D1**,**D2**) ascorbic acid content. CK represents the control group; T represents the enhanced UV-B treatment. A *t*-test was used to assess the significance of differences between T and CK at the same sampling time, with * for *p* < 0.05 and ** for *p* < 0.01.

The effects of the treatment on the main nutritional and flavour qualities of the fruit pulp are shown in Figure 3. TSS content in the fruit pulp of both the treatment and the control showed a consistent upward trend, and there was no significant difference between the two at each sampling time. TA showed a decreasing trend in the treatment group compared to the control during the later stages, resulting in a significantly higher TSS/TA ratio in treated fruits. This ratio exhibited a consistent increasing trend in both groups, with the treatment showing significantly higher values at later stages. ASA content in the fruit pulp showed a dynamic pattern, with an initial decline followed by a subsequent increase. Throughout this period, AsA levels in the treated fruits were consistently higher than those in the control. Combined with the above findings, these results suggest that the treatment enhanced the nutritional quality and flavour of the fruit by accelerating the maturation process.

### 3.3. Effect of Enhanced UV-B Radiation Treatment on the Physiological Damage of Mango Pulp

**Figure 4:**
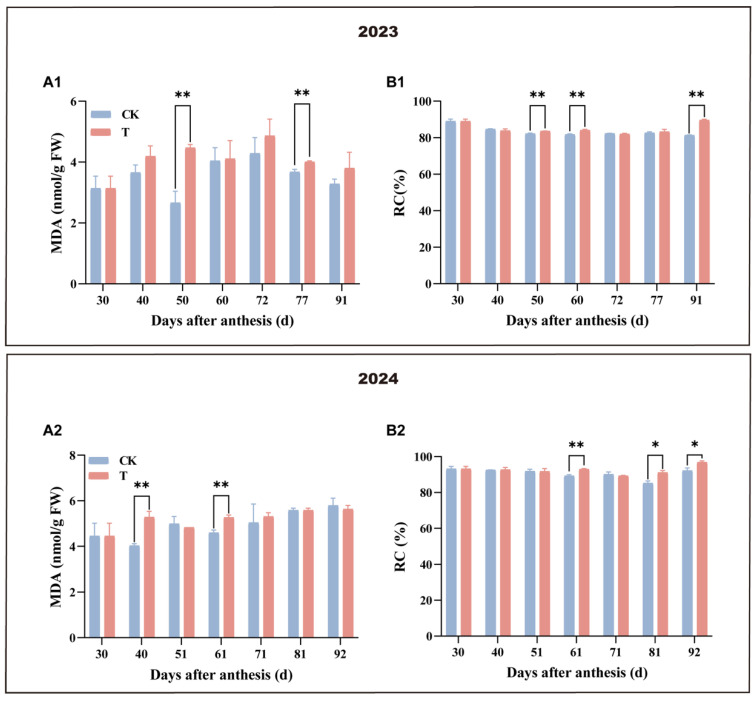
Effect of enhanced UV-B radiation treatment on the physiological damage of mango fruit pulp. (**A1**,**A2**) Malondialdehyde content (MDA); (**B1**,**B2**) relative conductivity (RC). CK represents the control group; T represents the enhanced UV-B treatment. A *t*-test was used to assess the significance of differences between T and CK at the same sampling time, with * for *p* < 0.05 and ** for *p* < 0.01.

The effects of the treatment on MDA content and RC in fruit pulp are shown in Figure 4. Over the past two years, MDA content showed slight differences between the treatment and control, with significant decreases observed 40 and 50 days after flowering in the control group, respectively. Notably, the MDA trends exhibited clear interannual variation between the two groups. The RC of both treatment and control exhibited a consistent trend of no significant change in two years. During the phase of rapid fruit expansion, both MDA content and RC were significantly higher in the treatment compared to the control. These findings suggest that the treatment induced membrane lipid peroxidation and increased membrane permeability, leading to smaller fruit size and earlier ripening that enhanced the flavour and nutritional quality of the fruit pulp.

### 3.4. Effect of Enhanced UV-B Radiation Treatment on the POD Activity and Lignin Content of Pulp

**Figure 5:**
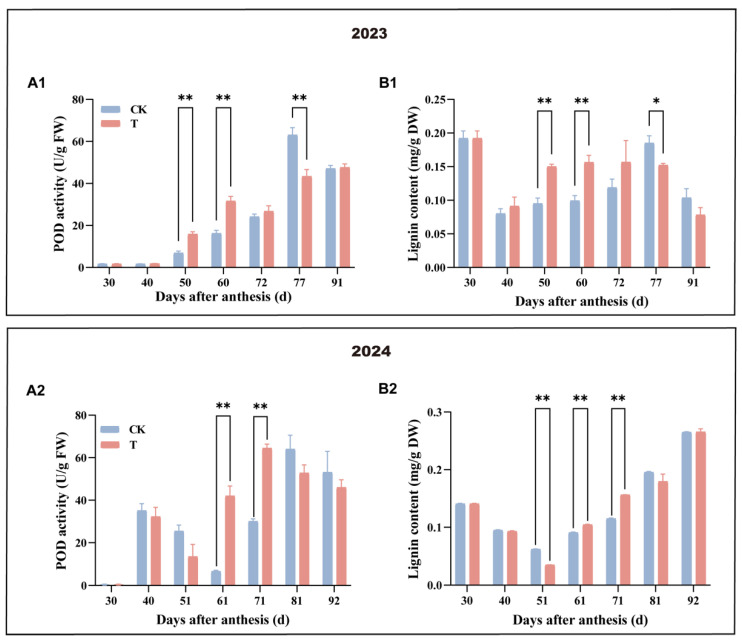
Effect of enhanced UV-B radiation treatment on the POD and lignin content in mango pulp. (**A1**,**A2**) POD activity content; (**B1**,**B2**) lignin content. CK represents the control group; T represents the enhanced UV-B treatment. A *t*-test was used to assess the significance of differences between T and CK at the same sampling time, with * for *p* < 0.05 and ** for *p* < 0.01.

As shown in Figure 5, the dynamic changes in POD activity and lignin content over two years exhibited fluctuations in both the treatment and control groups. During the mid-phase of rapid fruit expansion, the treatment showed significantly higher POD activity and lignin levels compared to the control. In contrast, the control group experienced a notable increase at harvest. These results indicate that enhanced UV-B radiation stimulated POD activity in the treatment, leading to greater lignin accumulation in the pulp and strengthening the fruit’s ability to withstand oxidative damage.

### 3.5. Effect of Enhanced UV-B Radiation Treatment on the Changes in Hormone Contents of Pulp

**Figure 6:**
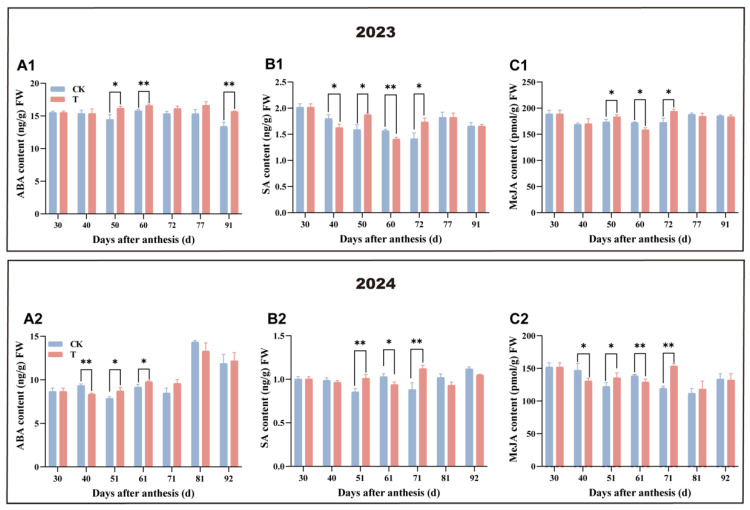
Effect of enhanced UV-B radiation treatment on the changes in hormone content in mango pulp. (**A1**,**A2**) Abscisic acid content, ABA; (**B1**,**B2**) salicylic acid content, SA; and (**C1**,**C2**) methyl jasmonate content, MeJA. CK represents the control group; T represents the enhanced UV-B treatment. The unit g in the figure refers to fresh weight. A *t*-test was used to assess the significance of differences between T and CK at the same sampling time, with * for *p* < 0.05 and ** for *p* < 0.01.

The dynamic changes in hormone levels between the treatment and control groups exhibited broadly similar trends across both years, with statistically significant differences observed at various developmental stages (Figure 6). Throughout the two-year period, comparable patterns of hormonal variation were detected between the treatment and control groups at corresponding sampling times. ABA levels in the treatment group were significantly higher than those of the control, 51 and 61 days after flowering in 2024. However, at 40 days, ABA levels in the treatment were significantly lower than those of the control. During the rapid fruit expansion stage, both SA and MeJA levels in the treatment group exhibited significant fluctuations compared to the control. The variation in hormone levels revealed distinct trends between the two years. In 2023, SA content in the treatment group was significantly lower than in the control at 40 days after flowering, whereas no significant difference was observed at the same stage in 2024. Conversely, MeJA levels in the treatment were significantly lower than in the control at 40 days after flowering in 2024, with no significant difference in 2023. At other sampling points, neither SA nor MeJA levels showed significant differences between the treatment and control groups. Despite these fluctuations, the overall trends remained consistent across both years, indicating that the treatment promoted earlier fruit ripening and reduced fruit size. These effects may be associated with elevated ABA levels during the mid-stage of rapid fruit expansion, as well as the variations observed in SA and MeJA content in the pulp.

### 3.6. Effect of Enhanced UV-B Radiation Treatment on POD Expression of Pulp

**Figure 7:**
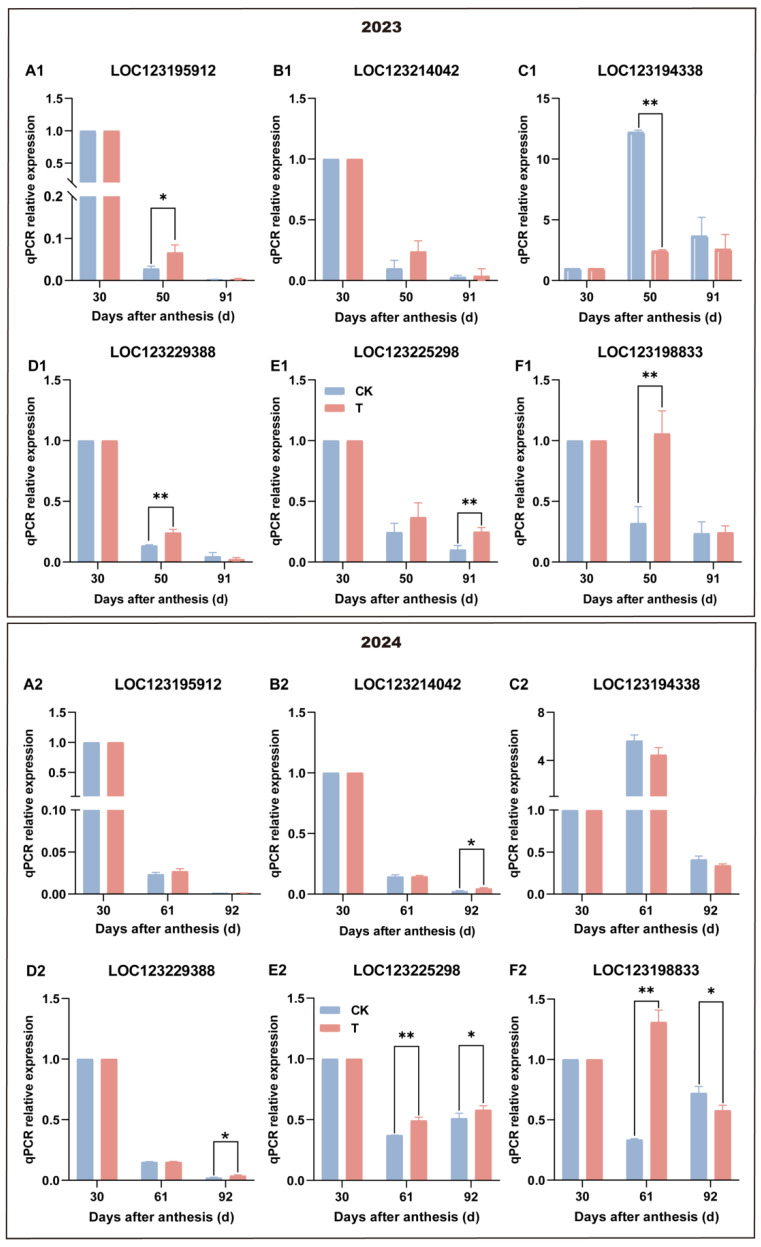
Effect of enhanced UV-B radiation treatment on POD expression in mango pulp. (**A1**,**A2**) *LOC123195912* gene; (**B1**,**B2**) *LOC123214042* gene; (**C1**,**C2**) *LOC123194338* gene; (**D1**,**D2**) *LOC123229388* gene; (**E1**,**E2**) *LOC123225298* gene; (**F1**,**F2**) *LOC123198833* gene. CK represents the control group; T represents the enhanced UV-B treatment. A *t*-test was used to assess the significance of differences between T and CK at the same sampling time, with * for *p* < 0.05 and ** for *p* < 0.01.

Six gene family members, including *LOC123195912*, *LOC123194338*, *LOC123225298*, *LOC123229388*, *LOC123214042*, and *LOC123198833*, were selected for two consecutive years, and the results are shown in Figure 7. In 2023, *LOC123195912* and *LOC123229388* exhibited significantly higher expression levels in the treatment group at 50 days after anthesis, while in 2024, *LOC123198833* and *LOC123225298* showed a similar upregulation at 61 days after anthesis. These expression patterns were consistent with the transcriptomic data. Overall, the results indicate that enhanced UV-B treatment induces the upregulation of key *POD* genes, which may enhance POD activity, promote lignin accumulation in the pulp, and help reduce oxidative damage by mitigating the effects of ROS.

### 3.7. Multivariate Linear Correlation Analysis

**Figure 8:**
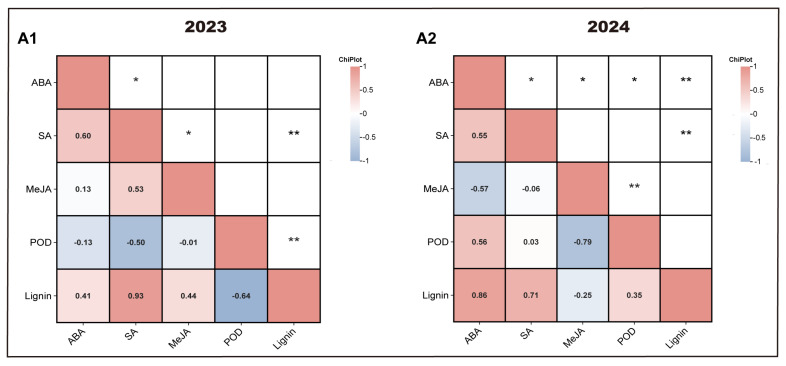
Multivariate linear correlation analysis of POD activity, lignin content, and hormone content. (**A1**) indicates 2023 and (**A2**) indicates 2024. CK represents the control group; T represents the enhanced UV-B treatment. A *t*-test was used to assess the significance of differences between T and CK at the same sampling time, with * for *p* < 0.05 and ** for *p* < 0.01.

Multiple linear correlation analyses were performed to investigate the relationships among hormone contents, POD activity, and lignin content in the pulp, with partial correlation coefficients presented in Figure 8. In 2024, a significant positive correlation was found between POD activity and ABA content, indicating that the treatment may have promoted POD activity by elevating ABA levels in the pulp.

**Figure 9:**
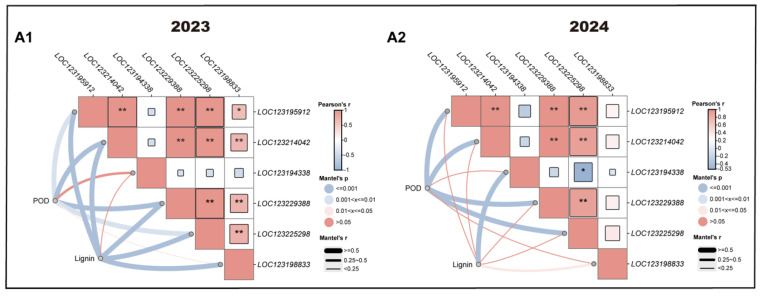
Multivariate linear correlation analysis of POD activity, lignin content, and expression of *POD* gene family members. (**A1**) indicates 2023 and (**A2**) indicates 2024. CK represents the control group; T represents the enhanced UV-B treatment. A *t*-test was used to assess the significance of differences between T and CK at the same sampling time, with * for *p* < 0.05 and ** for *p* < 0.01.

Similarly, multiple linear correlation analysis was carried out among POD activity, lignin content, and the expression levels of *POD* gene family members, as shown in Figure 9. In 2023, the expression levels of *LOC123198833* and *LOC123225298* were positively correlated with POD activity, while in 2024, *LOC123225298* continued to show a positive correlation. These results suggest that the treatment enhanced POD activity by upregulating these gene members, highlighting them as key contributors within the *POD* gene family that regulate POD activity dynamics.

## 4. Discussion

### 4.1. The Effect of Enhanced UV-B Radiation Treatment on the Fruit of ‘Tainongyihao’ Mango

Enhanced UV-B treatment caused chloroplast damage and reduced stomatal conductance in mango leaves, decreasing photosynthetic rate and resulting in reduced fruit weight per fruit [24,25,29]. This study observed consistent fruit size reduction effects; however, it also observed an improvement in internal fruit quality and earlier ripening, consistent with previous findings from our research group [27,30,31]. Hassam et al. elucidated the mechanism of reductive primary metabolites, revealing dynamic reprogramming of ascorbic acid (AsA) and glutathione (GSH) metabolism. This involved the upregulation of GSH-related enzymes (*GR/GST/GPX/G6PDH*) and genes (*MiGR/MiGPX*, etc.) coordinated with the downregulation of degrading enzymes (*GGT/MiGGT*), which collectively mitigated oxidative damage while potentially restricting fruit development [27]. Wei Ling et al. investigated flavonoid metabolic responses, demonstrating that upregulated key genes (*MiCHS7/MiCHI1*, etc.) drove the accumulation of monomers like quercetin, significantly enhancing antioxidant activity and nutritional quality while simultaneously reducing fruit size [30]. Shi Shaopu et al. explored the antioxidant enzyme response and found that upregulated peroxidase (POD) genes, along with increased lignin accumulation, significantly enhanced ROS scavenging capacity [31]. This mechanism effectively reduced membrane damage but suppressed cell expansion, ultimately leading to premature ripening and smaller fruit size. Furthermore, membrane lipid peroxidation during the rapid fruit expansion phase caused significant cellular damage, directly impairing cell division and elongation [25], unveiling the cytological mechanism underlying fruit size reduction.

The results of this study indicate that enhanced UV-B radiation treatment significantly increased POD activity in the pulp during the rapid fruit expansion stage compared to the control. This suggests that the treated pulp mitigated ROS damage, likely through the upregulation of POD activity, which is consistent with the previous research reports in both leaves and fruits [32,33,34]. The findings also demonstrated that lignin content was significantly higher in the UV-B-treated fruits compared to the control during the rapid fruit expansion stage. Lignin is capable of directly absorbing UV-B radiation [35,36], and, as a key phenolic compound, it plays a vital role in scavenging ROS [37]. These findings suggest that under enhanced UV-B radiation, mango pulp may mitigate oxidative damage and alleviate UV-B-induced stress through increased lignin biosynthesis. This adaptive response not only enhances the fruit’s tolerance to UV-B stress but may also contribute to improved resistance against UV-B stress.

### 4.2. The Mechanism of POD Activity Regulated by UV-B Radiation Treatment

Under UV-B radiation stress, ABA has been reported to alleviate ROS damage by enhancing the activity of antioxidant enzymes such as POD in several species, including grapevine (*Vitis vinifera* L.) and Yunnan poplar (*Populus yunnanensis* Dode.) [38,39,40]. Similarly, in wheat (*Triticum aestivum* L.), exposure to enhanced UV-B radiation increases POD activity in leaves by elevating the level of SA and MeJA, which collectively help reduce lipid peroxidation [41,42]. Consistent with these findings, our results also indicated that enhanced UV-B radiation treatment increases POD activity by elevating the levels of ABA, SA, and MeJA in the fruit pulp.

However, in a previous study, we identified 77 members of the POD multigene family (*MiPODs*) in the mango genome and performed a cis-element analysis of their promoter regions, and the key gene member of *MiPODs* induced by enhanced UV-B radiation was *LOC123195912*, screened through RNA-sequence and qPCR validation [31]. In this study, among these, six *MiPODs* members contained hormone-responsive cis-elements in their promoter regions. Specifically, ABA-responsive elements were found in *LOC123214042*, *LOC123194338*, *LOC123198833*, and *LOC123225298*; SA-responsive elements were present in *LOC123214042*, *LOC123225298*, and *LOC123195912*; MeJA-responsive elements were located in *LOC123195912* and *LOC123225298*; and antioxidant response elements (AREs) were present in *LOC123214042*, *LOC123194338*, *LOC123225298*, and *LOC123229388* [26]. Therefore, enhanced UV-B radiation treatment may upregulate the expressions of *LOC123198833* and *LOC123225298* by increasing the levels of ABA, SA, and MeJA in pulp, thereby enhancing POD activity; therefore, the more important gene member is not *LOC123195912* [31].

### 4.3. The Limitations of This Study

This study has certain limitations. The enhanced UV-B treatments were conducted under specific field conditions, without considering interactions with other climatic factors that could influence the physiological outcomes. Additionally, the focus on peroxidase (POD) limited the scope of understanding the antioxidant defence network, as other key antioxidant enzymes and non-lignin reduced components were not examined. Moreover, while transcriptome analysis identified candidate *POD* genes, their functional role remains to be validated through genetic manipulation, which is necessary to confirm their precise roles. So future studies integrating broader antioxidant pathways and functional genomics will be needed to fully elucidate the complex mechanisms of enhanced UV-B stress tolerance in mango.

## 5. Conclusions

During the rapid expansion stage of ‘Tainongyihao’ mango fruit, enhanced UV-B radiation significantly elevated the levels of ABA, SA, and MeJA in the pulp. These hormonal changes upregulated the expression of POD-related genes (*LOC123198833* and *LOC123225298*), leading to increased POD activity and lignin accumulation, which enhanced ROS scavenging. However, despite these protective responses, oxidative damage still occurred, disrupting mitosis and ultimately reducing fruit size. In the late stage of treatment, increased cellular injury was associated with a decline in POD gene expression and activity, potentially accelerating fruit ripening. These results reveal a dual-phase response mechanism in mango pulp under enhanced UV-B radiation and provide molecular insight into hormone-mediated regulation of antioxidant pathways.

## Figures and Tables

**Table 1:** Primer sequences for different genes.

Gene ID	Forward Primer Sequences (5′ to 3′)	Reverse Primer Sequences (5′ to 3′)	Expected Amplicon Size
*LOC123195912*	GGCGATGAAGACCCGTCT	ACTCCCGGGGTCCATCTC	108
*LOC123214042*	GTTGCACAAGCTTCCGGC	TCTTGGGCATCGACGTCG	107
*LOC123194338*	TTCGCTTGCTGGGGATGG	CCCCTGTCAAGGCAACGA	137
*LOC123229388*	GCTGCTCGTGACTCCGTT	TGCGGCGCTTAAACTTGC	93
*LOC123225298*	CTTCTGCAAGGTGGCCCA	TCTCGGCCGTAGCTTCCT	85
*LOC123198833*	CATGGACCCCGTCACACC	TGGCCCAGGCTTTTACGG	104
*Actin*	ATCTGCTGAAGGTGCTGAG	CCAAGCAGCATGAGATCAA	101

## Data Availability

The data that support the findings of this study are available from the corresponding author upon reasonable request.
